# Supplementary figures and images for: Network supporting contextual fear learning after dorsal hippocampal damage has increased dependence on retrosplenial cortex
Source: PLoS Comput Biol. 2018 Aug 7;14(8):e1006207. doi: 10.1371/journal.pcbi.1006207 (PMC6097702; doi:10.1371/journal.pcbi.1006207)

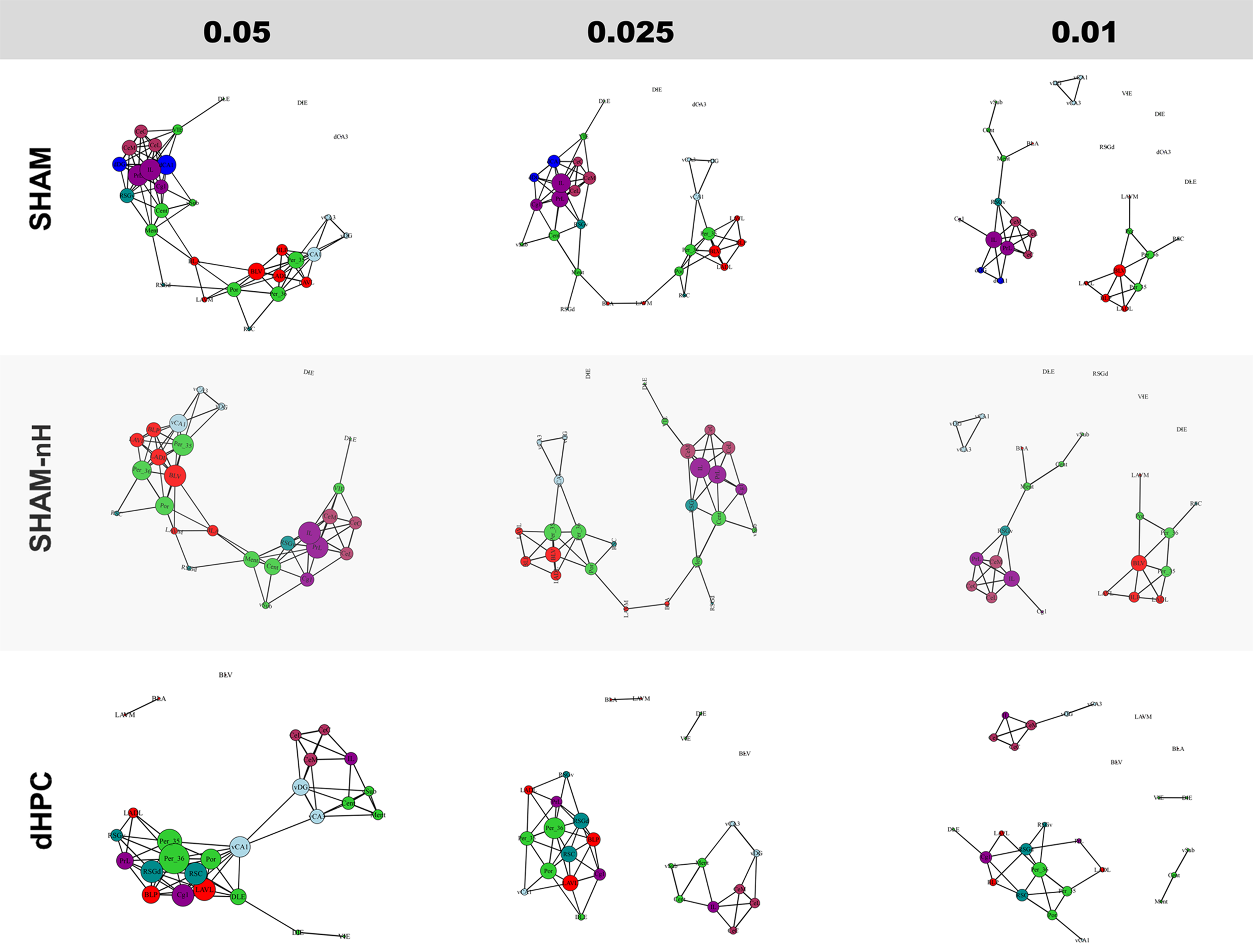

Supplement: S1 Fig — This network configuration reveals the regions that were more strongly correlated showing them closer to each other. The node sizes are proportional to the degree of each region. The edge width is proportional to its corresponding correlation coefficient. The networks are shown by group: SHAM (upper), SHAM-nH (middle) and dHPC (bottom); and by threshold: 0.05 (left), 0.025 (middle) and 0.01 (right). The node colors respect the code shown in Fig 2. (TIF) [file pcbi.1006207.s001.tif]

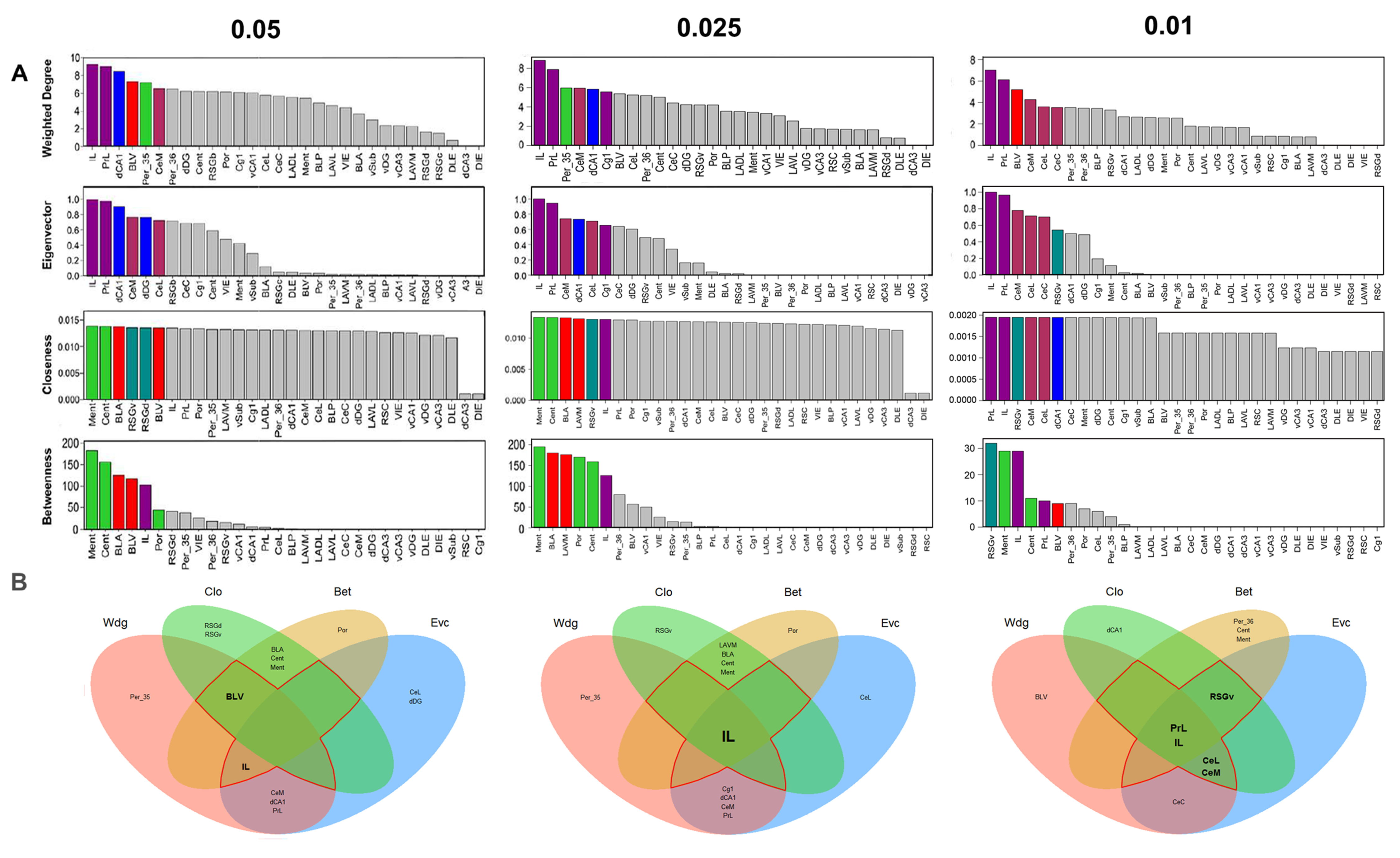

Supplement: S2 Fig — The centrality values were ranked according to each metric (A) and each threshold. Non-grey regions indicate the upper 25% in each metric. Regions are colored according to the color code on Fig 2. The intersection of the upper 25% most central regions in each metric (B) showed the hubs in each threshold, defined as regions present in at least three of the four metrics (red contour). (TIF) [file pcbi.1006207.s002.tif]

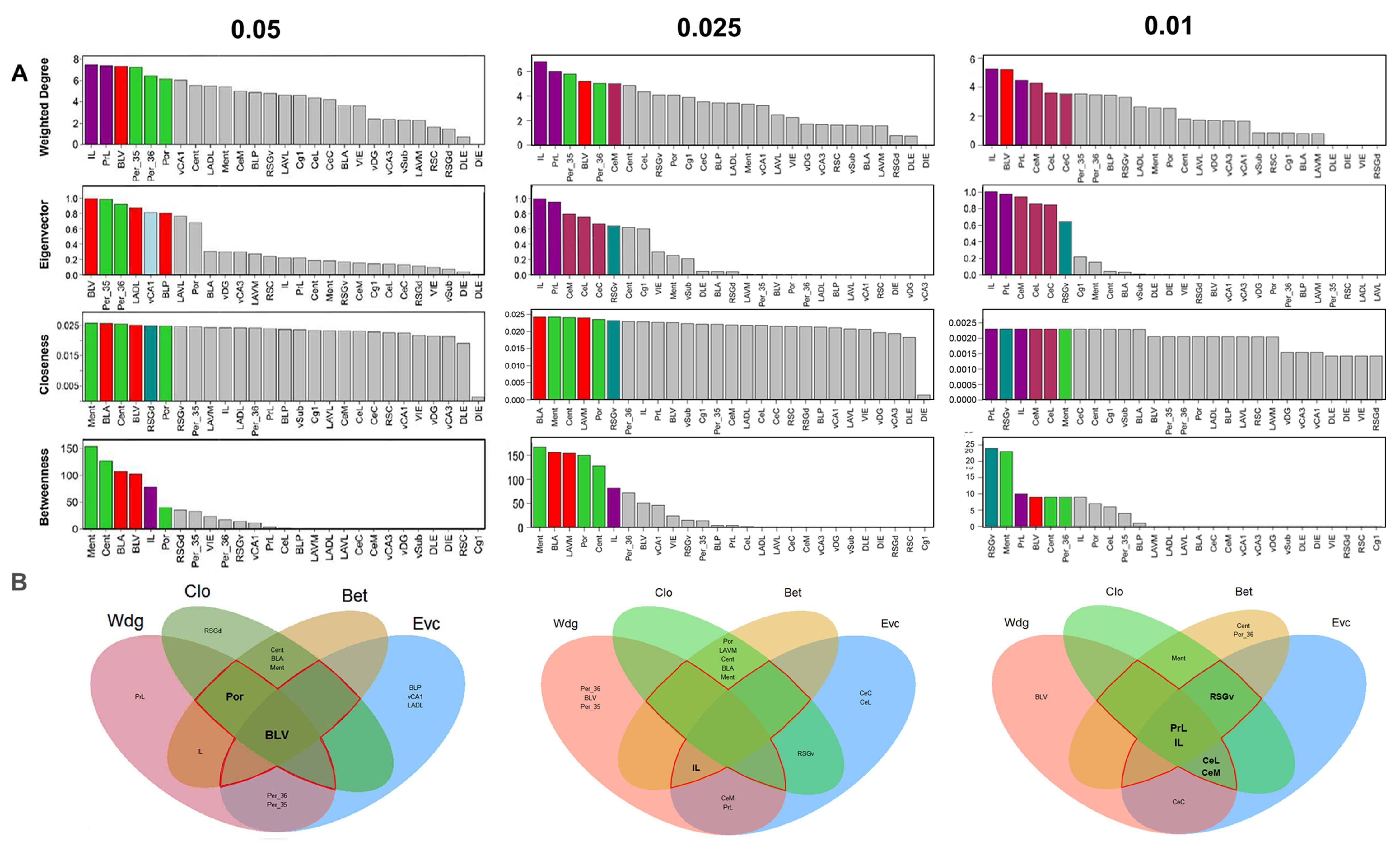

Supplement: S3 Fig — The centrality values were ranked according to each metric (A) and each threshold. Non-grey regions indicate the upper 25% in each metric. Regions are colored according to the color code on Fig 2. The intersection of the upper 25% most central regions in each metric (B) showed the hubs in each threshold, defined as regions present in at least three of the four metrics (red contour). (TIF) [file pcbi.1006207.s003.tif]

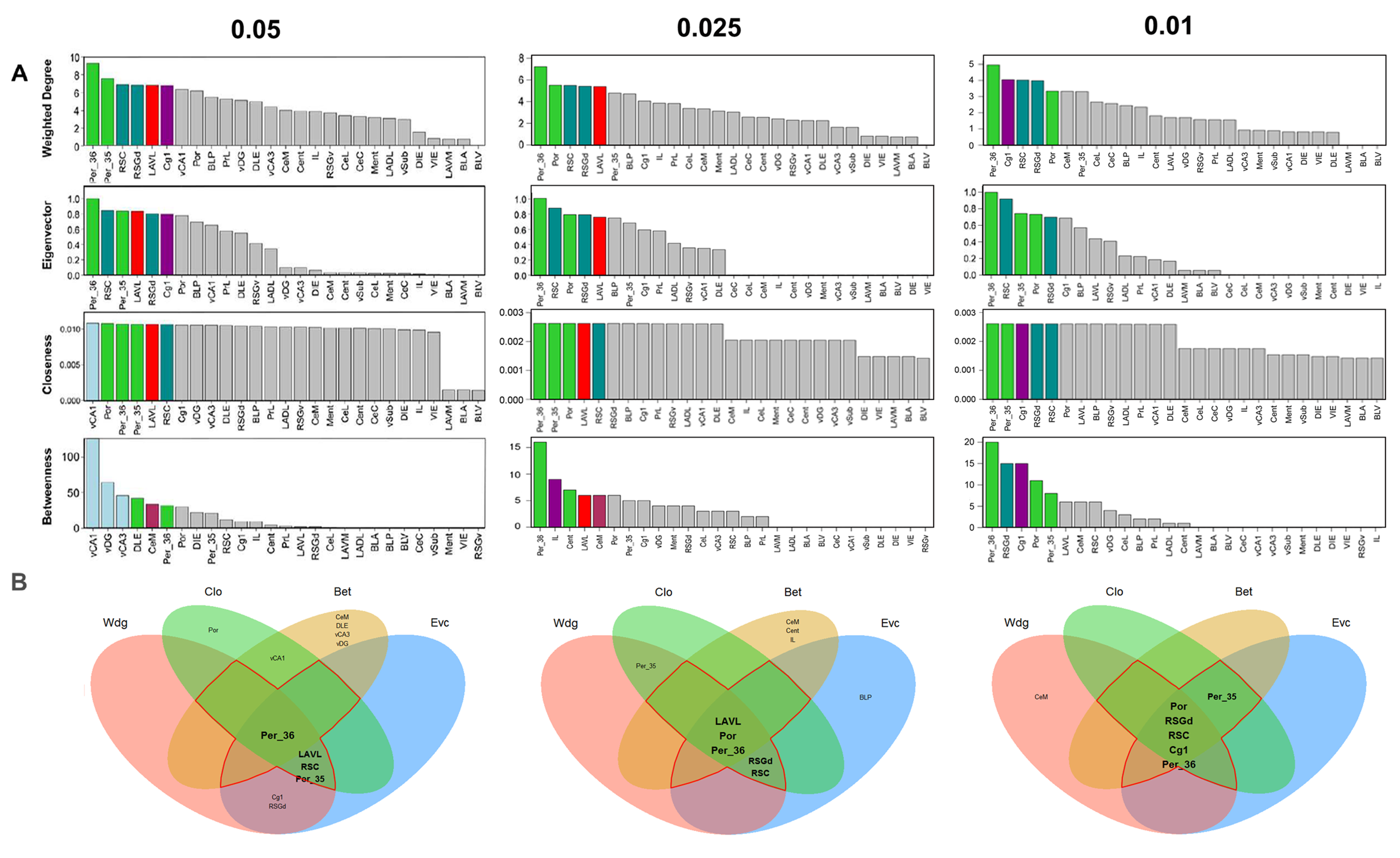

Supplement: S4 Fig — The centrality values were ranked according to each metric (A) and each threshold. Non-grey regions indicate the upper 25% in each metric. Regions are colored according to the color code on Fig 2. The intersection of the upper 25% most central regions in each metric (B) showed the hubs in each threshold, defined as regions present in at least three of the four metrics (red contour). (TIF) [file pcbi.1006207.s004.tif]

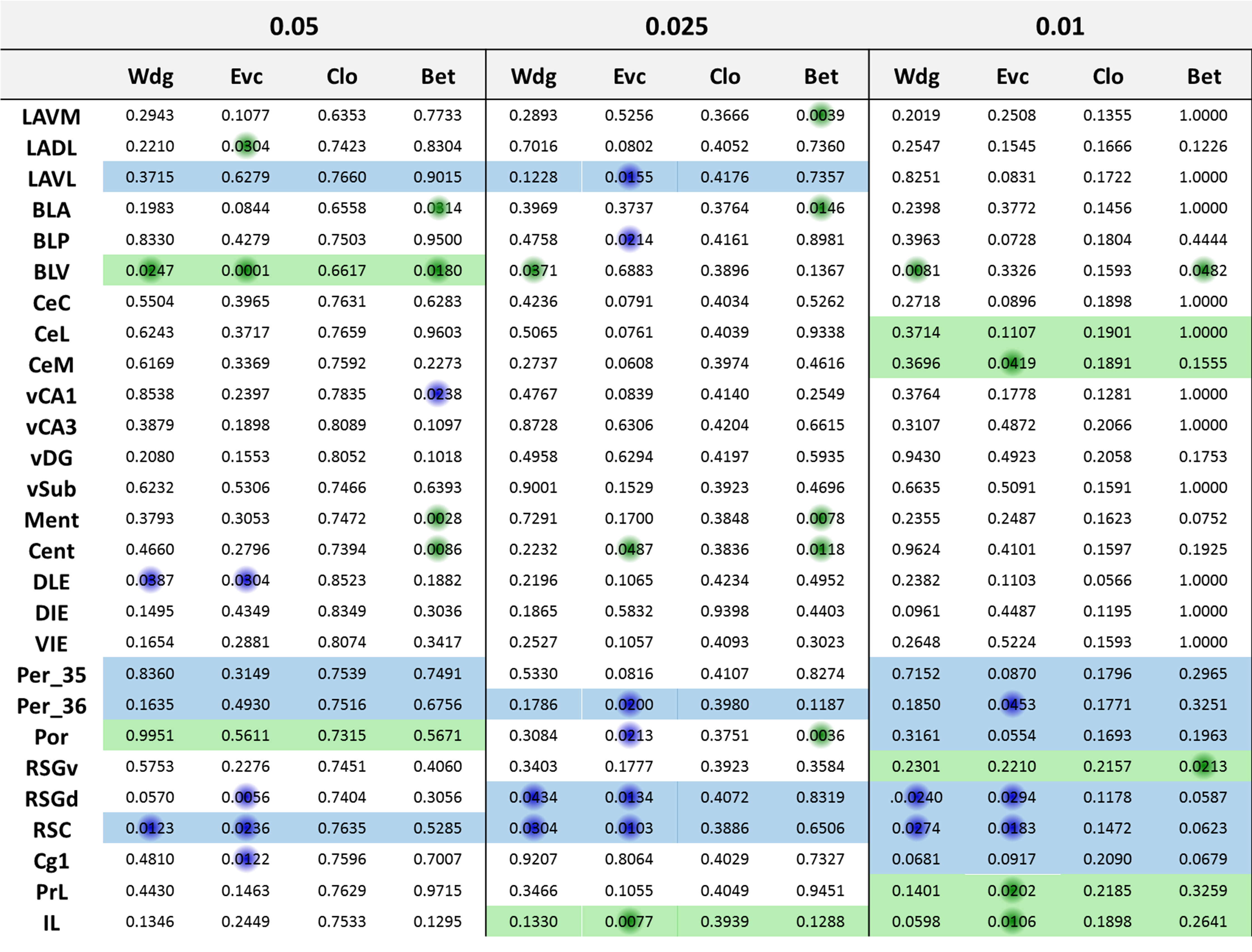

Supplement: S5 Fig — The comparison was done for each region, centrality metric and threshold. Values in each cell show the permutation test p-value for each comparison. Green round shades show significantly higher values in SHAM-nH, and blue round shades show significantly higher values in dHPC (p < 0.05). In each threshold, green lines indicate SHAM-nH network hubs for that threshold, and blue lines indicate dHPC network hubs. Values with lines and ground shades in the same color show hubs associated with significant differences. Wdg: Weighted Degree; Evc: Eigenvector; Clo: Closeness; Bet: Betweenness. This table is a detailed version of the Fig 7, where hub scores and differences can be seen by threshold. (TIF) [file pcbi.1006207.s005.tif]

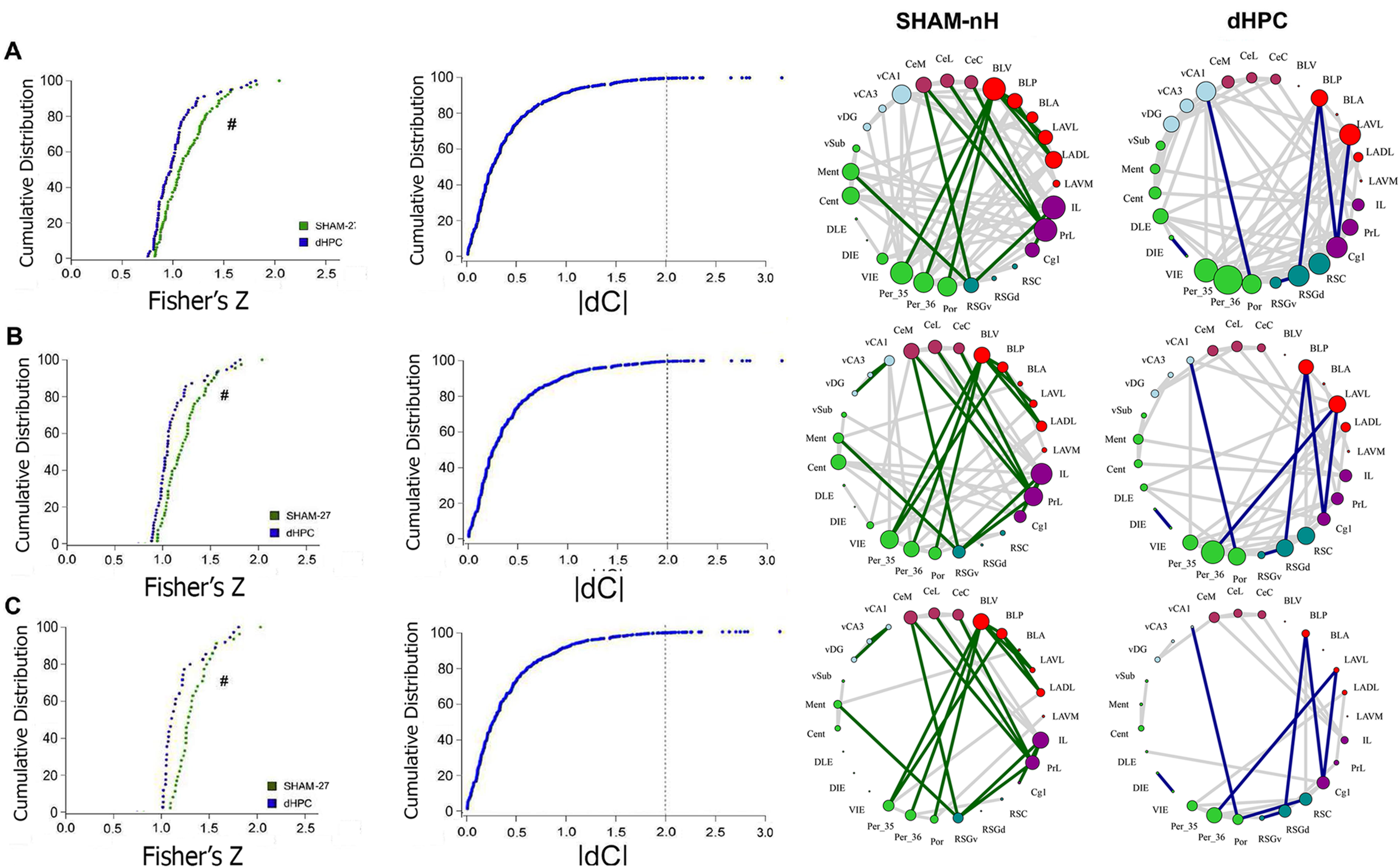

Supplement: S6 Fig — Cumulative distributions of the Fisher’s Z transformed correlation coefficients from the SHAM-nH and dHPC matrices (Left). The “#” indicates that these distributions are significantly different (Kolgomorov-Smirnov test, p<0.05). Cumulative distribution showing the z-score of the correlation coefficient differences between the groups (Center). The dashed line shows the absolute Z-score of 2, revealing the values considered significant (beyond it) at the level of α = 0.05. The significantly different coefficients were plotted in each network, showing the network and nodes to which it belonged (Right). Graphs shown for the networks in the 0.05 (A), 0.025 (B) and 0.01 (C) thresholds. (TIF) [file pcbi.1006207.s006.tif]

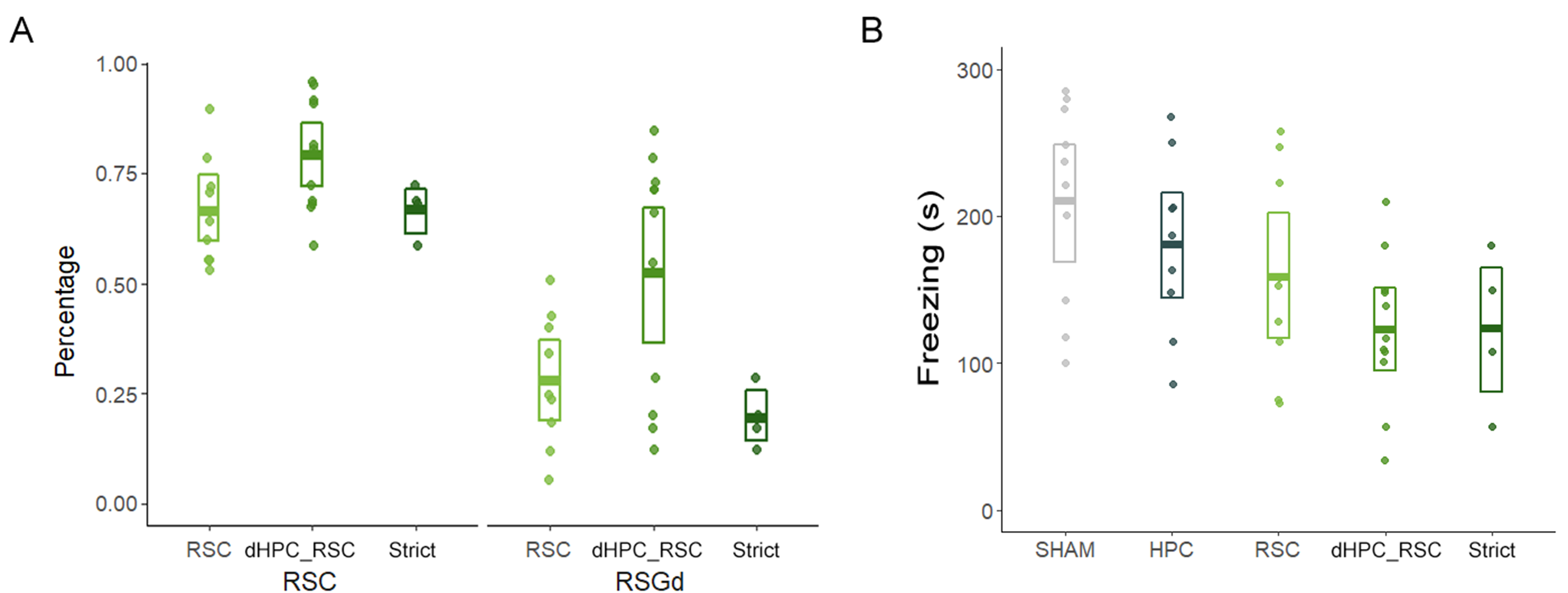

Supplement: S7 Fig — (A) Mean and bootstrapped 95% IC of the percentage of damage in the RSC and RSGd in the RSC and dHPC_RSC groups and in the strict dHPC_RSC subgroup. Dots show the sample distribution in each group. (B) Mean and bootstrapped 95% IC of the total freezing time in the SHAM, dHPC, RSC, dHPC_RSC groups and the strict dHPC_RSC subgroup. Dots show the sample distribution in each group. (TIF) [file pcbi.1006207.s007.tif]
