# Supplementary material for: Network supporting contextual fear learning after dorsal hippocampal damage has increased dependence on retrosplenial cortex
Source: PLoS Comput Biol. 2018 Aug 7;14(8):e1006207. doi: 10.1371/journal.pcbi.1006207 (PMC6097702; doi:10.1371/journal.pcbi.1006207)
Supplement: S1 Text — We describe two supporting experiments in which we show, in a within-subject design, a task that is impaired by dHPC damage followed by an unimpaired performance at CFC. These task were the post-training CFC and the water maze. These experiments replicate classical findings on the effect of dHPC damage on spatial/contextual learning and memory. (DOCX) [file pcbi.1006207.s008.docx]

**Network supporting contextual fear learning after dorsal hippocampal damage has increased dependence on retrosplenial cortex**

Cesar A.O. Coelho^1^, Tatiana L. Ferreira^2^, Juliana C.Kramer-Soares^1^, João R. Sato^2^, Maria Gabriela M. Oliveira^1^*.

^1^ Departamento de Psicobiologia, Universidade Federal de São Paulo - UNIFESP, São Paulo, SP, Brazil, zipcode 04023064

^2^ Centro de Matemática, Computação e Cognição, Universidade Federal do ABC, UFABC, São Bernardo do Campo, SP, Brazil, zipcode 09606070

**S1 TEXT**

Bellow we describe some experiments we performed to ensure the unimpaired performance of pre-training dHPC damaged rats in contextual fear conditioning. We carried two experiments that served as replication of previous studies that firstly showed that evidence as compared to tasks or manipulations in the dHPC that do result in memory impairment.

**MATERIAL AND METHODS**

***Subjects***

Forty-one male Wistar rats weighting 250-350g were obtained from the university vivarium (CEDEME, SP). They were housed in groups of 4 - 5 and maintained on a 12h light/dark cycle, room temperature of 22 ± 2^o^C, with free access to food and water. All experiments were approved by the University Committee of Ethics in Animal Research (#409649) and were in accordance with National Institutes of Health Guide for the Care and Use of Laboratory Animals.

***Surgery***

In the *S Experiment1*, the lesion method was identical to the procedure described for *Experiments 2* and *3* on the main text for the dHPC lesions (neurotoxic lesions by NMDA injections). In the *S Experiment 2*, the lesion method was the same as described for *Experiment 1* in the main text for the dHPC lesions (electrolytic lesions with anodic current). The postsurgical and recovery occurred just as described in the main text. In all experiments, there were only two groups: dHPC and SHAM.

***Apparatus***

For CFC experiments, we used the same apparatus described in the main text.

For the WM experiment, we used a black circular pool (2 m diameter and ~40 cm deep) filled with water at 23°C to a height of 25cm. We divided the pool in four quadrants, and placed a platform (10cm diameter) submerged ~2 cm below the water level in the ‘target’ quadrant. The platform was placed in a fixed region in the center of one of the quadrants. Distinct visual cues were positioned on each wall of the room for orientation. A camera installed on the ceiling and connected to a computer was used to record the behavior. We analyzed the behavior recorded using the Noldus EthoVision software version 7.0 (Leesburg, VA, USA).

***Contextual Fear Conditioning (CFC)***

All CFC procedures were identical to that described for *Experiment 1* in the main text.

***Water Maze (WM)***

The water maze task comprised a training phase and a memory test phase. The training consisted of 4 daily trials for 5 consecutive days. On each trial, we placed the rat in the center of one of the three non-target quadrants (pseudo-randomly chosen) and they had a maximum of 1 min to find the hidden platform. If the rat did not find the platform in 1 min, we guided them to the platform. Once in the platform, the rat remained there for 20 s before being transferred to a resting cage where he remained during the inter-trial interval (30 s). The behavior of interest during training was the latency to reach the platform.

Twenty-four hours after the last training day, the rats underwent a memory test. We placed each rat in the WM without the platform for 1 min. We recorded the time spent in each quadrant (target or non-target) by each rat.

***Statistical Analysis***

All analyses were performed in the same manner as the behavior analyses in the main text.

**RESULTS**

**S Experiment 1 – Retrograde but no anterograde amnesia of dorsal hippocampal lesion in contextual fear conditioning.**

In the *S Experiment 1*, we aimed to replicate previous findings regarding the effect of dorsal hippocampal lesions in retrograde and anterograde effects on CFC (Frankland et al., 1998; Wiltgen et al., 2006). The rats firstly underwent a CFC session and, 24 h later, the dHPC (N = 10) or SHAM (N = 9) lesion surgery. After the approximately 10 days of recovery, the rats underwent a contextual fear memory test (Test 1). Three days later, the same rats underwent a second CFC session and another memory test 48 h later (Test 2; **Fig 1A in S1 Text**).

A two-way ANOVA with bootstrap resampling showed a robust effect of group (F_1,34_ = 14.489, p = 0.0006) and of test (F_1,34_ = 7.523, p = 0.010), but no interaction between them (F_1,34_ = 1.755, p = 0.189). The p-corrected t-tests with bootstrap used for multiple comparisons showed an impaired freezing performance in the dHPC group on Test 1 (corrected-p = 0.001), but not on Test 2 (corrected-p = 0.430). There was no difference between tests for the SHAM group (corrected-p = 1.000), and a trend to a higher freezing time on Test 2 in the dHPC group (corrected-p = 0.065). The KS tests and Cohen D size effect showed the same pattern of results (**Fig1D in S1 Text**).


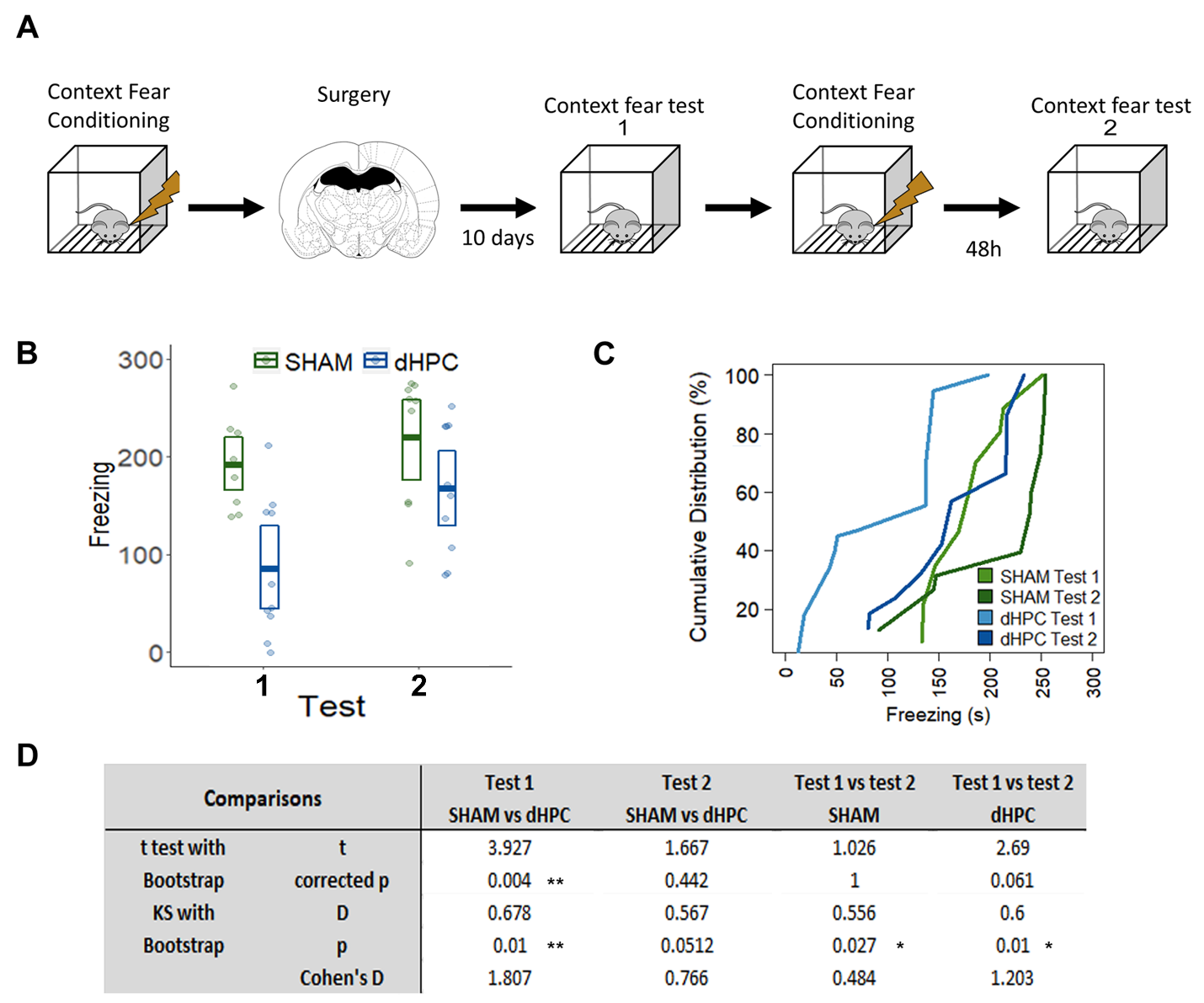


**Fig1 in S1 Text: dHPC lesion impairs CFC when occurred post-training, but not pre-training.**

(A) Experimental design of the *S Experiment 1*. (B) Mean (black line) and bootstrapped 95% CI of the mean total freezing time during the five min context fear memory test of dHPC (N = 10) and SHAM (N = 9) groups. The open circles show data distribution in each group. (C) Cumulative distribution of the sample as a function of freezing time showing the sample distributions. The “*” shows a significant difference between the groups (corrected-p<0.05). D) Table showing all the statistics and corrected p values of all comparisons pertinent for the study.

**S Experiment 2 – dHPC lesion impairs water maze learning and memory, but not contextual fear conditioning.**

In the *S Experiment 2*, we aimed to replicate findings from previous studies showing that pre-training dHPC lesions impair WM but not CFC (Richmond et al., 1999; Winocur et al., 2013). The rats first underwent dHPC (N = 11) or SHAM (N = 11) lesion surgeries. After recovery, they underwent 5 days of WM training, with 4 trials each day. On day 6, they were tested for spatial memory. Seven days later, the same animals underwent a CFC session, and their contextual fear memory was tested 48 h later (**Fig 2A in S1 Text**).

A two-way ANOVA with repeated measures and bootstrap resampling showed a robust group (F_1,100_ = 17.912, p < 0.0001) and day effects (F_4,100_ = 22.159, p < 0.0001), but no group x day interaction (F4,100 = 0.664, p = 0.622). These mains effects account for a higher latency to reach the platform in the dHPC group, although both groups do show learning over time (**Fig 2B in S1 Text**). The p-corrected t tests with showed that, in both groups, Latency on day 1 higher than on days 3 (SHAM: p = .0.003; dHPC: p < 0.0001), 4 (SHAM: p < .0.0001; dHPC: p = 0.017) and 5 (SHAM: p = .0.001; dHPC: p = 0.002). On WM memory test, a two-way ANOVA with bootstrap showed no main effect of group (F_1,84_ = 0.023, p = 0.880), but there was an effect of quadrant (F_1,84_ = 34.463, p < 0.0001) and a group x quadrant interaction (F_1,84_ = 11.285, p = 0.001). The p-corrected t tests with bootstrap showed a higher time of swim in the target quadrant in the SHAM group (p = 0.0003), but not in the dHPC group (p = 0.175). There was a tendency for a higher time in the target quadrant in the SHAM group compared to the dHPC group (p = 0.075). However, the t test with bootstrap did not show any group differences in the freezing time of the CFC memory test (t_20_ = 0.636, p = 0.531). The KS tests and Cohen D size effect performed for the memory tests confirmed these results (**Fig 2E in S1 Text**).

**
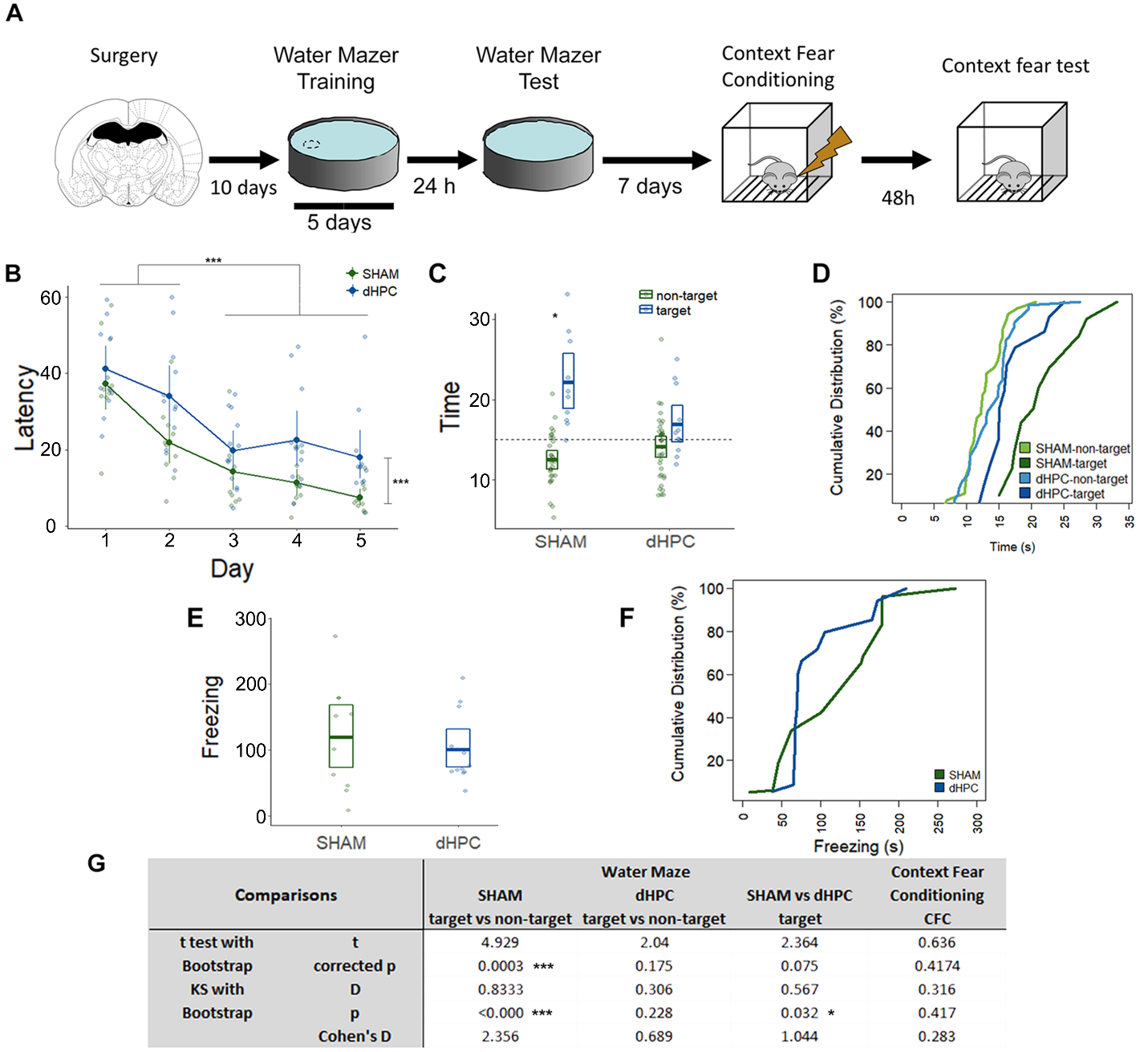
**

**Fig2 in S1 Text: Pre-training dHPC lesion impair WM but not CFC.**

(A) Experimental design of the *S Experiment 2*. (B) Mean (bigger darker dots) and bootstrapped 95% CI of the mean latency to reach the platform on the 5 days of training of both dHPC (N = 11) and SHAM (N = 11) groups in the WM. The smaller dots represent the distribution of the average of the four daily sessions. (C) Mean (darker, thicker lines) and bootstrapped 95% CI of the mean time swimming in the target or non-target quadrants during WM memory test. The lighter and smaller dots show data distribution in each group and day (or quadrant). (D) Cumulative distribution of the samples as a function of time swimming during WM memory test. (E) Mean (darker, thicker lines) and bootstrapped 95% CI of the mean freezing time during CFC memory test. (F) Cumulative distribution of the samples as a function of freezing time during CFC memory test. (G) Table showing all the statistics values and their corrected-p values of the comparisons made with the memory tests data. The “*” shows a significant difference (corrected-p<0.05). D) Table showing all the statistics and corrected p values of all comparisons pertinent for the study.

**REFERENCES**

Frankland PW, Cestari V, Filipkowski RK, McDonald RJ, Silva AJ (1998) The dorsal hippocampus is essential for context discrimination but not for contextual conditioning. Behav Neurosci 112:863-874.

Richmond MA, Yee BK, Pouzet B, Veenman L, Rawlins JN, Feldon J, Bannerman DM (1999) Dissociating context and space within the hippocampus: effects of complete, dorsal, and ventral excitotoxic hippocampal lesions on conditioned freezing and spatial learning. Behav Neurosci 113:1189-1203.

Wiltgen BJ, Sanders MJ, Anagnostaras SG, Sage JR, Fanselow MS (2006) Context fear learning in the absence of the hippocampus. J Neurosci 26:5484-5491.

Winocur G, Moscovitch M, Sekeres MJ (2013) Factors affecting graded and ungraded memory loss following hippocampal lesions. Neurobiol Learn Mem 106:351-364.
